# Adopting Mechanistic Molecular Biology Approaches in Exposome Research for Causal Understanding

**DOI:** 10.1021/acs.est.3c07961

**Published:** 2024-04-19

**Authors:** Amy L. Foreman, Benedikt Warth, Ellen V. S. Hessel, Elliott J. Price, Emma L. Schymanski, Gaia Cantelli, Helen Parkinson, Helge Hecht, Jana Klánová, Jelle Vlaanderen, Klara Hilscherova, Martine Vrijheid, Paolo Vineis, Rita Araujo, Robert Barouki, Roel Vermeulen, Sophie Lanone, Søren Brunak, Sylvain Sebert, Tuomo Karjalainen

**Affiliations:** † European Molecular Biology Laboratory & European Bioinformatics Institute (EMBL-EBI), Wellcome Trust Genome Campus, Hinxton CB10 1SD, U.K.; ‡ Department of Food Chemistry and Toxicology, University of Vienna, 1090 Vienna, Austria; § National Institute for Public Health and the Environment (RIVM), Antonie van Leeuwenhoeklaan 9, 3721 MA Bilthoven, The Netherlands; ∥ RECETOX, Faculty of Science, Masaryk University, Kotlarska 2, Brno 60200, Czech Republic; ⊥ Luxembourg Centre for Systems Biomedicine, University of Luxembourg, 6 avenue du Swing, L-4367 Belvaux, Luxembourg; # Université Paris Cité, Inserm unit, 1124 Paris, France; ▼ Institute for Risk Assessment Sciences, Division of Environmental Epidemiology, Utrecht University, Heidelberglaan 8 3584 CS Utrecht, The Netherlands; ∇ Institute for Global Health (ISGlobal), Barcelona Biomedical Research Park (PRBB), Doctor Aiguader, 88, 08003 Barcelona, Spain; ○ Universitat Pompeu Fabra, Carrer de la Mercè, 12, Ciutat Vella, 08002 Barcelona, Spain; ● Centro de Investigación Biomédica en Red Epidemiología y Salud Pública (CIBERESP), Av. Monforte de Lemos, 3-5. Pebellón 11, Planta 0, 28029 Madrid, Spain; □ Department of Epidemiology and Biostatistics, School of Public Health, Imperial College, London SW7 2AZ, U.K.; ■ European Commission, DG Research and Innovation, Sq. Frère-Orban 8, 1000 Bruxelles, Belgium; △ Univ Paris Est Creteil, INSERM, IMRB, F-94010 Creteil, France; ▲ Novo Nordisk Foundation Center for Protein Research, University of Copenhagen, Copenhagen, Blegdamsvej 3B, 2200 København, Denmark; ▽ Research Unit of Population Health, University of Oulu, P.O. Box 8000, FI-90014 Oulu, Finland

**Keywords:** Exposome, Molecular Biology, Toxicology, Human Health, Exposure, GxE, Environment

## Abstract

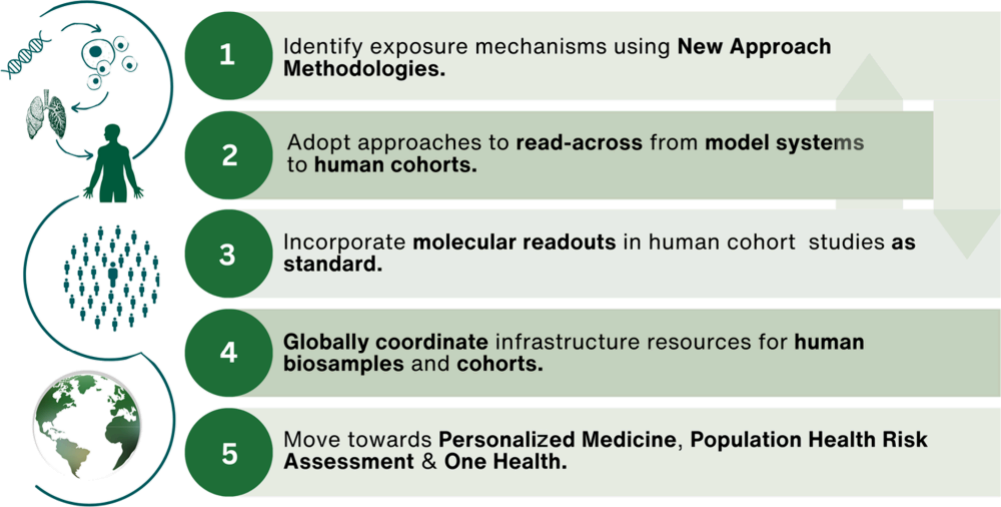

Through investigating the combined impact of the environmental exposures experienced by an individual throughout their lifetime, exposome research provides opportunities to understand and mitigate negative health outcomes. While current exposome research is driven by epidemiological studies that identify associations between exposures and effects, new frameworks integrating more substantial population-level metadata, including electronic health and administrative records, will shed further light on characterizing environmental exposure risks. Molecular biology offers methods and concepts to study the biological and health impacts of exposomes in experimental and computational systems. Of particular importance is the growing use of omics readouts in epidemiological and clinical studies. This paper calls for the adoption of mechanistic molecular biology approaches in exposome research as an essential step in understanding the genotype and exposure interactions underlying human phenotypes. A series of recommendations are presented to make the necessary and appropriate steps to move from exposure association to causation, with a huge potential to inform precision medicine and population health. This includes establishing hypothesis-driven laboratory testing within the exposome field, supported by appropriate methods to read across from model systems research to human.

## Abstract 2

This paper presents recommendations to integrate mechanistic molecular biology approaches in exposomics by bringing together expertise from an interdisciplinary research community.

## Introduction

The term “exposome” was first defined in 2005^1^ to capture the nongenetic factors in epidemiology. The exposome concept has promoted the systematic study of the environmental exposures (biological, chemical, physical, and social) that influence health, including the cumulative burden of exposure responses over time (e.g., allostatic load).^2,3^ In the last 10 years, uptake of the exposome concept in molecular epidemiology has led to the wider application of omics-based methods for the large-scale determination of associations between exposures and population-level health. Yet, identifying which associations are causal is complex. The integration of mechanistic molecular biology approaches within exposome research provides an unprecedented opportunity to elucidate molecular mechanisms for exposure effects, including growing potential in defining underlying gene–environment interactions (GxE) at cellular-to-population scales.

Written from a molecular epidemiology perspective, the seminal paper by Wild^1^ raised the potential for biomolecular approaches (genomics, transcriptomics, and proteomics) to define exposure impacts. Such approaches are now beginning to advance our understanding of the biological responses to exposures, not only through defining biomarkers but also pathways leading to disease phenotypes.^4,5^ This research is pressing considering the rise in preventable noncommunicable diseases (NCDs; e.g., cancer, mental illness, neurological, cardiovascular, and autoimmune diseases), which accounted for 90% of the premature deaths recorded in the EU in 2021^6^ and result from a complex GxE interplay. The necessity to capture molecular mechanisms underlying exposures, including GxE interactions, is widely acknowledged and the European Commission has allocated ∼€3 billion in environment and health research since 2000.^7^ However, the systemic adoption of molecular methods in epidemiology and exposome research is in its infancy and there is need for a roadmap to establish how biological information is integrated within the exposome field.^8,9^ Addressing exposome effects requires an interdisciplinary approach, and advanced molecular biology techniques have the potential to establish collaborations across data science, fundamental biology, environmental toxicology, chemistry, biotechnology, sociology, environmental science, genomics, and epidemiology to understand human health phenotypes. Building such an interdisciplinary research community requires the facilitation of knowledge exchange across fields with clear experimental guidelines, consistent terminology, and a robust, longstanding, international research infrastructure.

While the use of multiomics methods in epidemiology is now widely accepted, it constitutes only one, yet important, contribution of molecular biology to the exposome. In fact, molecular approaches can significantly aid in establishing causal, mechanistic relationships between exposures and health impacts (e.g., smoking and lung cancer^10^), particularly for phenotypes that result from complex and multifaceted exposures. Combining the holistic concept of the exposome with an in-depth mechanistic approach would be extremely beneficial to deciphering the underpinning processes of health trajectories. This review discusses approaches and recommendations to leverage such an interaction.

## A Call for Action: Integrating Biomolecular Approaches in Exposome Research

The multifactorial nature of the exposome challenges our ability to make direct associations between exposures and phenotypic outcomes.^11^ As such, the exposome has generally been studied in relation to three interconnected domains: (i) internal (e.g., from ingested chemicals), (ii) specific external (e.g., occupation, educational attainment), and (iii) general external (e.g., sociodemographic factors, air pollution). Since the emergence of the field in 2005,^1^ technological and methodological developments are helping to characterize exposures across these domains in greater detail, supported by advanced computational approaches that leverage data capital for the improved inference of phenotype-exposure associations.^12−14^

Novel approaches are required to overcome emerging barriers in identifying trends across fields and diverse data sources. These challenges can be broadly summarized into five key areas: *(i) incorporating social exposures*, challenges in capturing social exposure effects such as sociodemographic impacts and early determinants of health;^2,15^*(ii) analysis of multiomics data*, interpretation, cohesion, and comparison of multiomics data sets both within studies and between studies;^16^*(iii) statistical power*, exposome cohorts with omics readouts are generally small scale (<1k individuals), challenging the ability to identify statistically significant trends at the biomolecular level between exposure and effect; *(iv) deciphering GxE*, genotype is an essential and underpinning component of exposome impact, but as the exposome is multimodal in comparison to genotype, the increased complexity, variability, and dynamics of GxE interactions and a lack of a conclusive description of the exposome is a limiting factor in enabling such analysis;^16^*(v) defining generational impacts*, difficulties in distinguishing between genetic and nongenetic inheritance and a need for intergenerational cohorts.^17,18^

The following sets out the current progress and future steps for addressing these challenges using biomolecular approaches.

## Using Molecular Biology Techniques to Better Integrate Equity, Diversity, and Inclusion in Exposome Research

Recent research has emphasized the role of social inequalities as an overarching factor impacting health outcomes. Their influence, starting in early life, is supported by at least three streams of evidence in relation to health and disease: (a) the DOHaD theory (developmental origin of health and disease);^19^ (b) literature on Adverse Childhood Experiences (ACEs); (c) evidence on life-course socioeconomic disparities in health. ACEs have been associated with ischemic heart disease, obesity, perceived health, psychopathology, inflammation, mortality, health behaviors, and allostatic load later in life.

The onset of NCDs can be seen as the end point of a life-long trajectory of exposures that act via multistage mechanisms and express the biography of a person, with socioeconomic position throughout life being an overarching determinant of health.^20^ This highlights a pressing need to represent social inequalities in epidemiological studies as a determinant of health rather than a confounding factor (e.g., the Fundamental Cause Theory^20,21^). This is similarly related to the concept of “weathering” which is used to describe the consequences of structural discrimination (such as ethnicity and gender) on health.^22^ Establishing more accurate and specific data collection on social inequalities and social structure will enable more meaningful associations with molecular markers. Current data collection on structural social inequalities and social stratification in epidemiological studies has been very simplified. This is even more difficult in low-to-middle income countries (LMICs), where social structures tend to be more complex and difficult to capture with the tools usually applied in Western populations.

However, there are increasing opportunities to incorporate social epidemiology within exposomics, for example by connecting human cohorts with administrative records (e.g., the Administrative Data Research UK program^23^), geographic indicators and, more recently, historic records (e.g., air quality and deprivation indices^24^). In the case of social exposures, where quantitative measures cannot be obtained, consideration of errors, bias, and uncertainty, and methods such as weight-of-evidence scores are beginning to be implemented.^25−27^ Population and health research is biased toward Western populations, which significantly impacts the strength of findings in a global context (e.g., as highlighted in genomics^28^). The challenges associated with moving toward equality in health research are multifaceted and include a reduced ability to record specific external factors in LMICs due to high costs of personal monitoring and supporting infrastructures, compounded by poor public health resources. While measures of general environmental factors can be facilitated at a global scale (e.g., via satellite monitoring) and provide indicators of general exposures, the ability to understand responses experienced at the individual level by specific and internal exposures is challenging. Lessons can be learned from population-health genomics which has benefited those of European ancestry but, due to a lack of genomic diversity, fails to provide the same benefit on a global scale.^29^

Investigations into the intermediate mechanisms that mediate biological responses to social experiences have begun in fields such as epigenetics,^30,31^ through understanding mechanisms of age-acceleration (DNA-based clocks)^32^ and biomarker discovery for allostatic load.^32−34^ This mechanistic research gives a biological basis to the concept of the “embodiment” of social inequalities.^35^ Capturing the influence of negative early exposures on health throughout life has significant implications for informing positive interventions in public health policy and, for example, promoting investment into various forms of social, economic, and cultural capital in childhood. Capturing social exposures in a systematic way will provide opportunities for identifying mechanisms associated with social exposure effects.^36^

We recommend that social circumstances, including inequity, structural racism, and social exclusion, should be incorporated into exposome research as an integral component, leading to physiological and pathological changes that can also be traced through internal biomolecular changes. This concept, also expressed as the “embodiment” of social relationships, requires novel approaches to data collection and study design. The current collection of information on social circumstances in epidemiology is rudimentary, and an improvement requires strong collaboration with social scientists. A common misconception is that social inequalities (frequently and incorrectly named “SES”, socioeconomic status) are a confounder of causal relationships. The use of internal markers to investigate the propagation of social circumstances into the body is a useful but also expensive approach that is not necessarily feasible, for example, in LMICs. This calls for a refinement of other types of tools imported from the social sciences with better discriminatory and predictive power compared to questionnaires. Molecular tools—like “epigenetic clocks”^37^—can be used to better clarify the causal pathways, to provide biological plausibility, and to validate other types of nonmolecular evidence.

## Bridging Omics in Exposome Research

The application of omics-based molecular techniques in human cohort studies is now commonplace, providing quantitative biomolecular readouts in large cohorts (e.g., from blood, saliva, and fecal samples^38^) through to clinical settings (e.g., tumors and tissue samples). An increasing number of exposomic research approaches rely on omics technologies to characterize responses to exposures^8,9,39^ which provide opportunities to capture gene (transcriptomics and epigenomics^40−42^) to protein-level responses (proteomics^31^) as well as define internal microbial community composition (metagenomics^32^) and metabolic perturbations.^43−47^ Examples include identifying associations across >100 different exposure types (social, chemical, and lifestyle) during pregnancy with DNA-methylation changes in children,^5^ providing opportunities to investigate adverse childhood exposures on health phenotypes in later life. These data sets can also be supported and analyzed in the context of recent frameworks defining the exposome and mixture effects acting via eight core biomolecular mechanisms as outlined by Peters et al;^4^ ensuring data sets for each hallmark are established in cohort studies provides opportunities to define mixture effects.

Technological advances in mass spectrometry (MS) analysis and quantitative characterization of chemical exposure agents in field and biosamples^22^ have been particularly beneficial to exposome research. It is now possible to track chemicals and their transformation products over time both in the environment and individuals at increasingly lower concentrations using targeted and nontargeted (NT) approaches.^48−51^ Nontargeted approaches hold promise for extrapolating the more comprehensive measures of chemical exposure agents and their metabolites in biosamples to mixture dosages more representative of real-world exposure. However, challenges exist in analyzing these results against existing chemical libraries with most features remaining undefined. The standardization of methods will assist in strengthening NT-MS in exposomics further, and guidance is being developed toward this aim.^52^

As the use of omics increases, this highlights challenges in identifying the biomolecular responses to specific exposure source(s) and parameters (e.g., exposure route(s) and time period, environmental vs endogenous effects) from cohort exposure data alone (e.g., defining impacts of air pollution from satellite data). Readouts provide a snapshot of the biological responses at tissue or cellular levels, reflective of sampling time postexposure, and are likely to be affected by confounding factors, such as external physiological cues (light, temperature, and seasonal variation^53^), which form part of the exposome but complicate the understanding of specific exogenous stressors.

The interpretation of static readouts therefore requires knowledge of biomolecular pathway cascades and effects across the cellular-to-whole-organism trajectory, including how responses are spatially and temporally interlinked. Integration of molecular measures and analysis of mechanisms such as gene-regulatory networks, cell-to-cell and interorgan communication (particularly through the blood) will go some way to achieve this and begin to reveal biomarkers reflective of an exposome-induced propensity to disease.^2,54^

Multiomics data sets from longitudinal human cohorts are beginning to provide new opportunities to establish more substantial and grounded evidence of biomolecular responses to exposures,^3,5,55−58^ but conducting omics profiling (metabolomics, genomics, transcriptomics, proteomics, microbiome) is limited by financial constraints. Predicting the total cost of complete biomolecular profiling per sample and the feasibility of omics at scale is dependent on multiple factors, including whether samples are analyzed in industry vs academic settings as well as the availability of commercial kits or providers. Metabolomic profiling of blood and urine provided by Nightingale Health is one example^59^ (with costs starting from <€30 per sample), whose clients include the UKBioBank, where nuclear resonance mass-spectrometry (NMR) has been used to profile 249 metabolomic biomarkers across all 500k UKBB samples.^60^ By comparison, MS-based metabolomic profiling platforms are diverse with costs varying depending on factors including sample preparation, analyte coverage, and the precision of quantification. While nontargeted screening assays cost <€100 per sample, for targeted analysis, costs can vary from €20 to €2k per sample depending on the analytes measured and approach used. The decision to choose between nontargeted and targeted screens is dependent on the scientific question. While costs will decrease as technologies continue to develop, there is a need to prioritizse readouts of interest and work collectively and across fields to define key biomarkers indicative of exposure effects that could be profiled at scale.

Currently, two clear study designs have been developed in relation to the application of omics in the exposome space: (1) to understand phenotypic responses over time and (2) to identify population-level effects. Studies with repeated, and ideally multigenerational, measures using a single omics technique are currently far more valuable than multiomics analysis at a single time point with respect to understanding phenotypic change. However, single time-point multiomics analysis provides a different understanding of associations e.g. between levels of biological organization (cellular to protein to metabolic). In both instances, there is a need to cross-reference cohorts to increase the statistical power. In (1) this can be done by comparing an individual response across time points against a continuous variable and in (2) by comparing individuals to a wider population using the population as a reference baseline.

**Figure 1:**
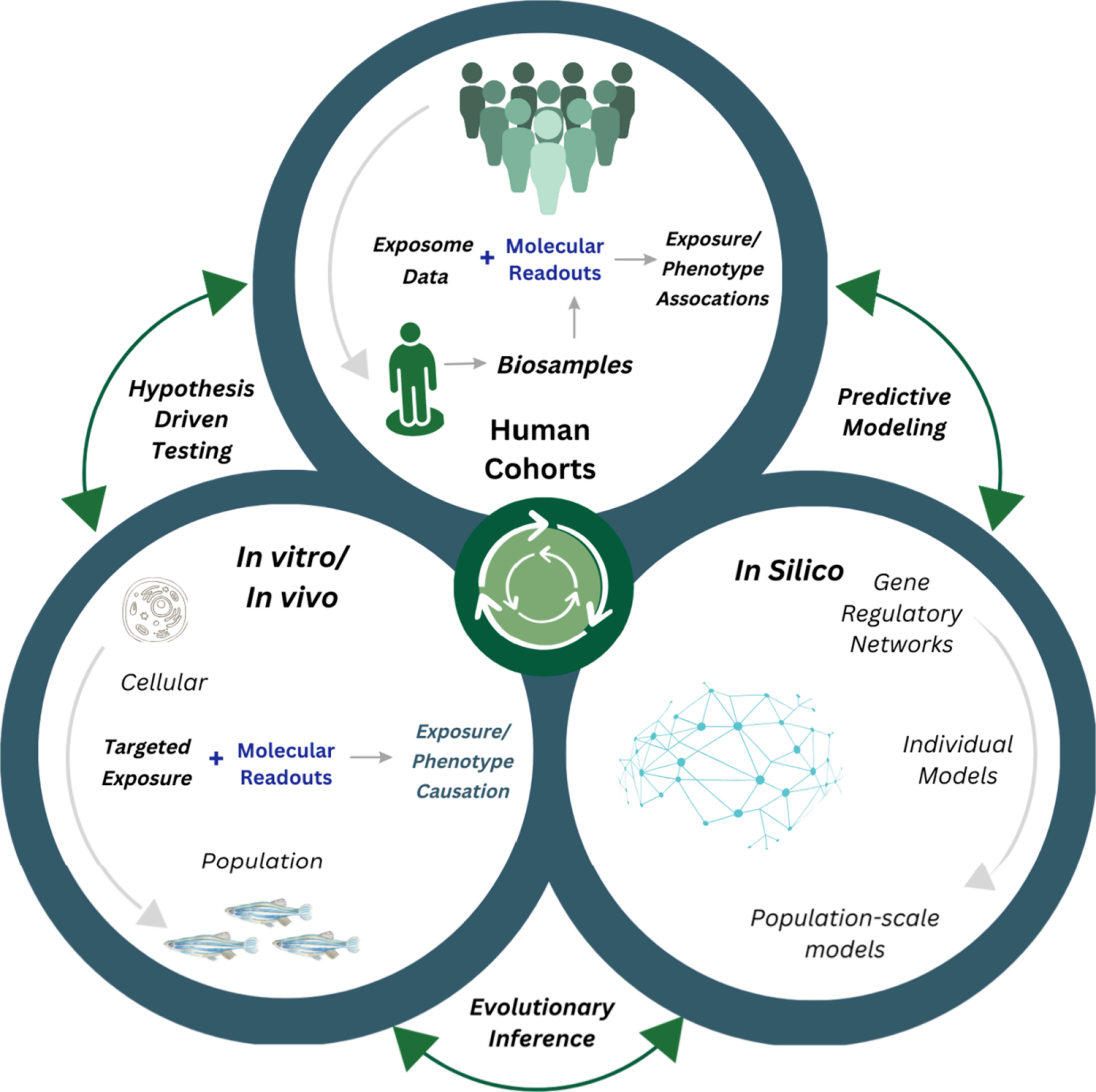
Integrating molecular biology approaches into exposome research. Integrating the analysis of human cohort data with molecular techniques to identify exposure causation and GxE through informing hypothesis driven secondary testing *in vitro* and *in vivo* using model systems. Wet lab methods are supported by *in silico* techniques, from cell to population levels (e.g., gene regulatory networks to predictive population exposure risk models). Evidence of effects is compiled in a knowledge repository (such as an Adverse Outcome Pathway model).

With the cost of multiomics a limiting factor, hypothesis-driven *in vitro*/*in vivo* studies based on omics associations identified in exposome cohorts are not only cost-effective but vital to providing further evidence of suitable molecular biomarkers as proxies of both exposure and affect that could be profiled at scale (Figure 1). As the field develops, there are opportunities to retrospectively leverage existing omics data from cohorts to maximize exposure-causation with the support of outputs from experimental studies providing evidence to interpret exposure outcomes in specific time frames.

## Opportunities to Integrate Exposome Research and Population Level Genomics

The ability to characterize genotype-specific and/or individual responses to exposures is particularly relevant to the future application of exposomics in predictive GxE models for precision medicine, population-scale monitoring, and resilient health care systems.

Whole-genome sequencing in population-scale biobanks such as Iceland (deCODE),^61^ FINNGEN,^62^ UK BioBank,^38^ and the Danish National Cohort Study (DANCOS)^63^ has led to the development of a number of techniques to identify cause–effect relationships between genetic signatures and phenotypes including genome-wide association studies (GWAS),^64^ phenome-wide association studies (PheWAS),^65^ and forward/reverse Mendelian randomization.^66^ For example, these methods have allowed for the characterization of rare genetic variants associated with health phenotypes such as the eye^67^ and cardiovascular diseases.^68^ In clinical cohorts, genomic data sets have been used to characterize mutational signatures underlying complex NCDs such as cancer.^69^ Using GWAS, polygenic risk scores (PGSs)^70,71^ are being adopted to predict relative likelihoods that an individual will acquire a disease based on genetic background alone. While the application of PGS in clinical settings is beginning to be realized internationally with the development of associated infrastructure resources,^72^ the full indication of risk and potential routes of intervention cannot be successful without an understanding of environmental exposure influences.^71,73−75^ This is particularly true for NCDs, where genetic variance is predicted to account for only a small share of disease risk.^73^

Characterizing human phenotypes based on cause–effect relationships between the exposome and genomic variability is a vital step that will benefit substantially from the integration of genomics and exposome research fields. The application of newer omics technologies to large biosample collections is providing unprecedented insights into human health, for example single-cell sequencing of blood samples in population-scale biobanks;^76,77^ these data sets can be advantageous to the exposome field through the immune-profiling of blood cells as an indicator of an individual’s past exposure to infectious agents. Qualitative proxies of exposures (for example, dietary self-reporting, smoking, and mental health questionnaires in the UK BioBank^38^) exist in such cohorts which are beginning to be leveraged to identify GxE associations for single-exposure interactions e.g. identifying the impact of childhood tobacco exposure and polygenic-risk scores on cancer incidence.^78^

There are growing opportunities to leverage national prescription registries and health record data to define GxE. In the absence of genetic data, longitudinal records of prescriptions alone can provide insights into optimizing drug selection in precision medicine,^79,80^ though this faces challenges in data interpretation.^81^ Current efforts to compile administrative and health records such as in the UK (ADRUK)^23^ have potential to be integrated with genomic data (e.g., through UKBB and genomics health records) and are a growing area of potential for exposome research. Such a focus would be beneficial, considering that medical prescription records are well-documented across Europe.

While cohort studies can provide evidence for a GxE association, *in vitro* studies (e.g., organoid or other) are required to address the pathways involved in the interactions (e.g., if a genotype decreases metabolism of a xenobiotic, then the latter may have larger effects^82^).

## The Application of Hypothesis-Driven Secondary Testing in Exposomics

Due to the complexity of the exposome, it is not possible to ascertain (1) causal relationships with multiple exposures and (2) GxE relationships from the biomolecular analysis of human cohort studies alone. It is therefore necessary and appropriate to provide further evidence for environmental-molecular effect associations through defined, hypothesis-driven research in closed systems. The exposome community must learn and apply expertise from other fields to better model human health exposure risks at the molecular level.

The application of existing research evidence into a human exposome context, for example, from fundamental research into epigenetics^83^ or from environmental toxicology,^84^ could provide an additional avenue for strengthening causal determination.

In the context of GxE, the in-depth genomic profiling of health phenotypes to gain mechanistic understandings using appropriate model systems (for example, microbial exposures in humanized mice^85^) provide opportunities for interactions between genotype and controlled exposures of environmental relevance to be identified (Figure 1). Such research, informed by the prioritization of responses from epidemiology studies to functional biology-driven screens, will allow for the identification of exposures with the greatest detectable effects.

One such avenue of untangling GxE from cohort studies is through identifying the interaction between PGS, widely used to define genotype–phenotype relationships, and the exposome.^86,87^ In a toxicology context, human-induced pluripotent stem cells (iPSCs) represent a new approach methodology (NAM) to reduce animal testing in human toxicology screens (in this case, secondary laboratory testing using iPSCs and the models derived from them (e.g., organoids)). With emerging opportunities to derive primary cells from a range of biosamples (e.g., peripheral blood mononuclear cells relevant to the immune responses, hair keratinocytes, and urine^88^ that can subsequently be reprogrammed to differentiate into multiple cell types (e.g., urine-derived iPSCs can be reprogrammed to urinary epithelial cells, endothelial cells, nerve cells, skeletal myogenic cells, osteoblasts, adipocytes, and chondrocytes^88^)), this provides a promising opportunity to test GxE.

Research should focus on the specific biological pathways and processes in the cell types associated with the PGSs established for the phenotypes of interest, which can be validated through the analysis of molecular and cellular end points (e.g., gene expression and functional assays). Replicating experiments across multiple cell lines and assays will ensure robustness and reproducibility of the findings and representativeness across the population. Finally, comparing the results with omics readouts from population cohorts will establish the implications of biomolecular effects, bridging the gap between *in vitro* findings and alignment with population-level disease prevalence. Stem-cell-derived models (e.g., organoids) are a promising approach but requires overcoming major limitations in the reproducibility and scaling (e.g., establishing protocols for reprogramming and differentiation) as well as improving financial accessibility to allow for high-throughput testing.^89^

In the past decade, advances in molecular biology have led to the development of a range of tools and high-throughput methods that have allowed the adoption of molecular techniques in the laboratory analysis of environmental exposure risk. This includes structural biology, where methods have been applied to assess ligand-binding affinities of environmental pollutants and associated biological receptors as indicators of molecular mechanisms of action for subsequent downstream effects.^90^ Such research provides supporting evidence for safe-by-design chemicals with lower impacts on human and environmental health.

Technological advances such as expansion microscopy^90^ are allowing high-resolution images with increased granularity to decipher cellular processes and protein dynamics.^91^ When coupled with immunostaining methods targeting proteins or microbial communities, such evidence on spatial localization is establishing new insights into internal human microenvironments and exposure interactions.^92^ In line with this, computational methods have advanced across the sciences, enabling the deployment of artificial intelligence (AI) and machine learning (ML) to model biological systems, compounds, and complex molecule-to-phenotype pathways by incorporating evidence with increasingly complex parameters.^93,94^ These developments provide opportunities to understand the exposome using experimentally tractable methods relevant to humans and able to capture real-life combinational responses.

From a genomics perspective, the advent of CRISPR-based tools allows high-throughput screens of gene-function through gene editing in cell lines and model organisms. Such systems can be used to secondarily test genetic trends identified in human cohorts, for example, for obesity where rare homozygous variants identified in human cohorts have been established using *Drosophila melanogaster* as a model system.^95^ In addition, the advent of self-organizing stem-cell-based models (organoids) has allowed for patient-derived tissues of specific organs to be partially recapitulated in the laboratory to understand GxE interactions.^87^ Such human organoids are more physiologically relevant to humans than animal models, and the impact of environmental perturbations can be ascertained under defined experimental windows and across a variety of genetic backgrounds.

## Understanding Molecular Mechanisms Underlying Exposures in Toxicology

While the majority of exposome research aims to identify the influence of environmental exposures (chemical, biological, physical, and social) on health using human cohorts, there is an opportunity to gain a mechanistic understanding of exposures at the molecular level by building on advances in toxicology, with promise for better understanding and modeling adverse health outcomes related to the exposome^96^ (Figure 1). Recent publications highlight synergies between exposome and toxicology research with compelling reasons to forge collaborations by bridging relevant information across different levels of biological organization.^96^ This is demonstrated through the successful integration of mechanistic molecular knowledge from epidemiological approaches and experimental evidence from molecular *in vitro* assays in the risk assessment of endocrine disruptors.^97−99^

The toxicology field is already leveraging the benefits of molecular techniques to ascertain the mechanisms underlying single chemical compound exposures in human health, where a reductionist approach is taken to drive chemical risk assessment and regulation. The adoption of molecular methods in high-throughput systems has identified common signatures and biomarkers of affected molecular pathways underlying environmental exposure responses. These approaches are beginning to provide avenues to determine causal relationship between exposures and health outcomes (for example, phenol/phthalate effects in pregnancy^97^).

More recently, the movement toward NAMs aimed at establishing nonanimal models for human toxicology testing holds promise for exposome research.^100^ Access to, and the study of, precise human molecular pathways *in vivo* (e.g., tissue specific, dose–response relationships) is limited by ethical, financial, and technical factors. NAMs overcome these challenges by providing opportunities to understand molecular pathways through *in vitro* models, including stem cell lines and organoids as well as *in silico* approaches such as machine learning techniques, computational systems biology, and agent-based modeling.^101^ The movement toward NAMs could create new scientific paradigms in exposome research by characterizing the mechanistic pathways underlying observations in human cohorts^97−99^ across stressors by enabling (i) high-throughput testing and comparison of omics readouts *in vivo* and *in vitro*, (ii) data integration and analysis through the use of ML/AI to identify experimentally defined responses in large human cohort data sets, and (iii) establishing biomarkers of exposures to aid in the identification of potential risk factors in epidemiological studies. Importantly, testing strategies using a combination of NAMs have been shown to be better predictors of adverse effects in humans than animal models alone,^101^ particularly for complex end points such as brain development.^102^ These experimental systems are starting to form the basis of “Integrated Approaches for Testing and Assessment” (IATAs), which combine multiple sources of experimental evidence to map mechanistic effects from genotypic to population levels^103^ (Figure 1).

Research initiatives aimed at establishing animal-free risk assessment methods for human toxicology testing, such as the ASPIS Cluster^104^ of Horizon Europe projects and the PARC European Partnership,^105^ could provide further opportunities and frameworks for identifying exposure impacts. This includes developments in comparative toxicology (as initiated by the Horizon Europe funded PrecisionTox^106^), which is beginning to inform risk assessment approaches by grouping chemical toxicants based on shared outcome responses across animal phyla. Such research is highlighting not only how pathways of toxicity are known to be conserved but that many exposure agents operate via the same pathway (i.e., relatively nonspecific).^107^ This provides opportunities for the application of nonmammalian animal models used in fundamental biomedical research to define the molecular mechanisms of exposures (e.g., *Drosophila melanogaster*, *Danio rerio*, *Caenorhabditis elegans*) including for social stress^108^ and diet.^95^ Such research should help to prioritize hypothesis-driven laboratory testing from exposome cohorts and has implications for identifying exposures of concern across environmental to human health perspectives.

## Developing Frameworks for Integrating Molecular Biology and Exposomics

**Figure 2:**
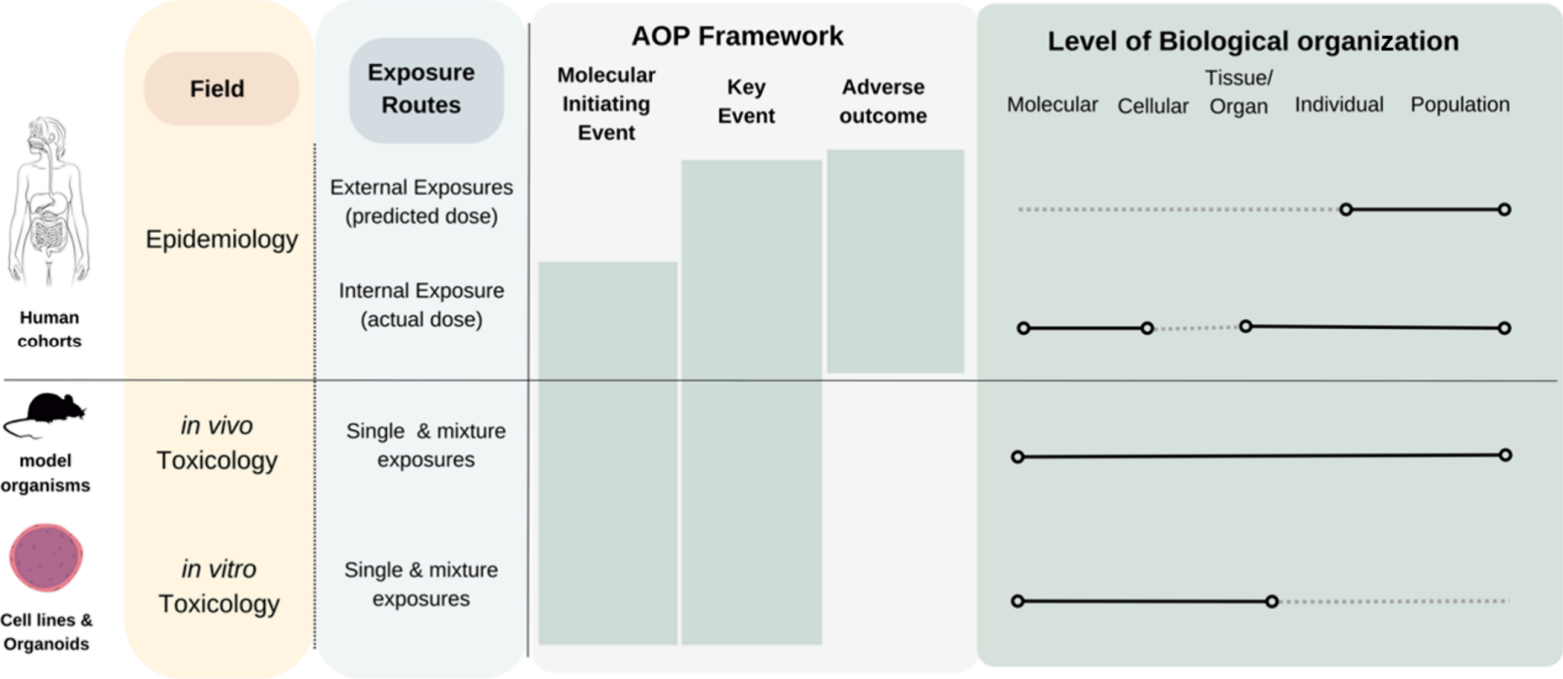
Integrating evidence across biological scales. Molecular Initiating Events (MIEs) including processes such as receptor activation/antagonization, oxidative stress, and DNA/protein and enzyme interactions can be determined *in vivo* and *in vitro*. Through measuring responses to exposures using omics and imaging techniques (e.g., histology and immunohistochemistry), Key Events (KEs) can then be described leading to the AO. In humans, longitudinal biomonitoring of selected cohorts can be used to determine responses to exposures, the mechanism of which can be supported by model-organism research.

Integrating information from various disciplines to unravel how exposures affect health outcomes is complex, requiring clear frameworks to interpret experimental results across scales and different levels of biological organization^5,6^ (Figure 2). As such, there are opportunities to leverage established approaches in toxicology within the exposome context to facilitate the transfer of knowledge from mechanistic studies. Defined as linear cascades, adverse outcome pathways (AOPs) provide one option by organizing mechanistic knowledge on overt adverse health effects (adverse outcomes) at individual and population scales^109^ from *molecular initiating events (MIEs)* through to *key events (KEs)* at cellular, tissue, and organ levels. As an agnostic conceptual framework, AOPs allow for the interpretation of the sequential molecular evidence of exposure impacts across stressors (social, chemical, biological, and physical).^96^ This makes the framework particularly relevant to exposome science as different types/combinations of stressors can influence the same key event (KE) or AOP. The adoption of AOPs in exposome research could enhances the utility of mechanistic molecular data^110^ by

1. Informing a mechanistic understanding of adverse effects
2. Examining the relative contributions of various components of the exposome on health phenotypes
3. Determining the primary risk drivers under multiple exposures.

The proposal for “life-course” AOPs to account for the allostatic load of social, physical, and chemical exposures experienced throughout life could enable a framework for networks of interactions between KEs influenced by different stressors to be defined in a time-dependent manner.^20^ This would allow for a holistic understanding of how adverse outcomes result from multiple exposures. However, while collaborations with toxicology and molecular biology fields are essential to establish mechanistic evidence *in vitro*/*in vivo* (Figure 2), this presents several challenges.

This includes researching molecular responses to multiple exposures in a single study. For example, studying effects of complex chemical mixtures still has many limitations such as accurate mixture reconstruction in laboratory testing (lack of standards, limited information on real-world compositions, complexity of exposure sequences,^87^ lack of suitable data exchange formats) and current frameworks for assessing risk, largely established in chemical and drug toxicity testing, lack additional information, for example, exposure route in pharmacokinetic models (e.g., dermal vs ingested, which impacts dose and elimination route). Coordinating, interlinking, and building on developments in toxicology (such as in ONTOX^111^ and PARC^105^), may begin to allow for characterizing complex mixture effects in detail, particularly when coupled with advances in exposome characterization as discussed above. There are also opportunities to couple AOPs with aggregate exposure pathways (AEPs) to define the impacts of mixtures on shared molecular targets.^112^

Overall, molecular pathways identifying how an exposure might affect a health outcome (such as AOPs) can be used to inform exposome studies and build hypotheses about toxicologically relevant chemicals and mixtures to study specific health outcomes. Single compounds and mixtures can then be studied using relevant NAMs to investigate hypotheses and assess causality. Knowledge of pathways and underlying molecular mechanisms will strengthen biological plausibility, helping with the selection of relevant biomarkers in exposome studies, informing epidemiological analyses of the exposome–health associations. Such approaches have been implemented, for example, to address bisphenol A and analogues.^98^

## Recommendations toward a Molecular Future in Exposome Research

A clear roadmap to integrate advanced molecular techniques in exposome science is necessary to gain a better understanding of the causative mechanisms underlying the exposure effects. This must take advantage of the current strengths and opportunities in the exposome field by building on existing infrastructure frameworks and research networks. It also requires addressing challenges such as creating cohesive terminologies across disciplines and in designing and capturing human cohort data consistently to allow for interoperability across data sets. There are three main outcomes from a close collaboration between the exposome and molecular biology that are critical for the development of functional exposomics:^2,8^ (1) using and exploiting multiomics in epidemiological studies; (2) improving knowledge on GxE interactions; (3) using experimental and computational studies to improve the evidence on causal associations between exposure and effects.

Our vision is to develop a connected community of scientists who address exposome research questions through dynamic interdisciplinary collaborations across fields such as epidemiology, toxicology, public and environmental health, bioinformatics, and the biomedical and social sciences. The impacts would be enhanced by robust, scalable research infrastructure and funding opportunities that are responsive to the interdisciplinary, interconnected nature of exposome research. Moving away from classical single-exposure single-outcome approaches, research funding in the past decade has reflected the value of cross-cutting, integrative approaches to understand adverse or protective effects of multiple exposures over various life periods.^7^ These efforts build on current funding approaches headed by the European Commission, including the European Human Exposome Network (EHEN),^113^ a cluster of nine research and innovation action projects, and subsequent research infrastructure support through the European Strategy Forum on Research Infrastructures (ESFRI) to form the Environmental Exposure Assessment Research Infrastructure (EIRENE).^114^ This program of work has established the EU as a global leader in exposome research, further supported by the initiation of a Horizon Europe coordination and support action in 2024 to leverage an International Human Exposome Network (IHEN); this network will be linked with similar efforts in the US National Institutes of Health (NIH) call 2024 to promote and advance international cooperation in exposome science.

There is a need to develop approaches to better understand the spatiotemporal effects of mixed, multifaceted exposure types (e.g., combined impacts of exposures in urban environments such as physiological impacts of air pollution and psychological responses to a lack of green space) rather than single agent/factor responses in isolation. It is essential that the growing community develops strong links with risk assessment organizations and policy makers to effect change and deliver on the full potential of its discoveries.

The following recommendations outline an interdisciplinary vision for exposome research whereby the identification of the molecular mechanisms underlying the responses to exposures are prioritized in a move from exposure association to causation.

### Underpin Exposome Research with New Approach Methodologies to Identify Molecular Mechanisms

NAMs provide opportunities to characterize the molecular responses to exposures at a level that is inaccessible in human cohorts. Moving from identifying exposure associations to causation using these systems employed in fundamental biology and toxicology would strengthen the evidence underlying exposome research in policy and governance. This is particularly true in the context of AOPs, where if several exposures are linked to the same AO, NAMs provide an opportunity to study KE relationships allowing for (i) high-throughput testing and comparison of omics readouts, (ii) data integration and analysis including through the use of ML/AI to interpret large data sets and identify trends in epidemiology readouts, and (iii) establishing biomarkers of exposures and exposure effects to aid in the identification of potential risk factors in epidemiological studies. The use of NAMs to define the effects of the exposome will be particularly beneficial for the following.

#### Noncommunicable Diseases

These require the need to identify mechanisms underlying complex mixture and exposure effects. Such research will involve building on current toxicology methods that investigate chemical mixture responses and expanding to account for additional exposure parameters such as social stress, diet, temperature, and underlying genetics.

#### Intergenerational and Transgenerational Effects

These require analyzing impacts across multiple generations by adopting model organisms with short lifespans (e.g. *Drosophila melanogaster*, *Caenorhabditis elegans*, and *Danio rerio*). Measuring GxE is essential to understanding phenotypic change, yet, it is challenging to differentiate between the genetic and nongenetic inheritance of traits. The use of biomodels provides the opportunity to disentangle inherited gene regulation (e.g., epigenetics) and other nongenetic impacts (e.g., social learning of behaviors to represent cultural transmission).

#### GxE Interactions

These can be best studied using high-throughput screens in cell lines and stem-cell-derived models (organoids) by screening single exposures and mixture effects on models with a variety of different genomic backgrounds. Patient-derived organoid models allow exciting opportunities to identify responses to exposures in the context of genetic modifications associated with disease outcomes, particularly for tumorigenesis. The development of integrated organoid models and microfluidic devices creates additional opportunities to study factors such as interorgan communication (e.g., combining vasculature and brain organoids to model the blood–brain barrier) in human-relevant contexts.

While approaches to investigate complex mixtures are beginning to be adopted in environmental toxicology, more is needed to link such experimental frameworks with the exposome field with clear applications to human health research. This requires developing experimental exposure systems synergistic to real-world human health exposure scenarios through both dosing and route of uptake (e.g., dermal, ingestion, inhalation). There is a need to standardize experimental exposure scenarios to ensure they are comparable across research institutions. For example, the feasibility of multisite operation of a standardized GC-HRMS assay to profile chemicals in human blood is being assessed via the EIRENE analytical QA/QC working group. There is also the need to establish protocols for deriving iPSCs.^89^ Possibilities also exist to begin developing proxies of exposure mixtures (e.g., air pollution mixtures in urban environments from epidemiological evidence) that could be used for *in vitro*/*in vivo* mixture studies.

### Incorporate Molecular Readouts in Human Cohort Studies as Standard

There is a need to build on work in coordinated biobanks (e.g BBMRI-ERIC^115^) to collect consistent, appropriately preserved biosamples from human cohorts (e.g., blood, saliva, fecal samples). The standardization of subsequent omics methods and analysis to community defined guidelines and in accordance with Findable, Accessible, Interoperable, Reuseable (FAIR) principles^116^ will begin to allow for more coordinated cross-comparison between studies and with hypothesis-driven secondary testing. Data sets would allow for the systematic analysis of omics readouts with opportunities to identify markers of both exposures (e.g., through profiling for chemical toxicants and their metabolites using mass spectrometry) and their impacts (e.g., biomarkers of molecular response pathways). However, it is important to acknowledge that such biomarkers will only provide a snapshot of the molecular responses at the time of sampling, the response to which will be dependent on multiple factors including exposure type, dose, age, genomic background, and allostatic load background of the individual.

### Adopt Approaches to Read-Across from Model Systems to Human Cohorts

Given the complexity of exposures, there is a need to harness computational methods that have the potential to bridge experimental evidence across epidemiological data sets and laboratory assays. The ability to integrate data sets generated using model systems (recommendation 1) through to omics readouts from human cohorts (recommendation 2) is required to establish causation and identify conserved response pathways through biomarkers and omics techniques. In this regard, AOPs provide a useful framework, as they allow the identification of relevant exposure biomarkers using NAMs relevant to human health, thus providing an evidence-based comparison of experimental and human data. The international collaborative effort to identify and develop AOPs and AOP networks such as the AOPwiki^117^ and to develop guidelines that allow for the transfer of AOPs mostly developed through experimental work to human health should be increased.

### Create Globally Coordinated Infrastructure Resources for Human Biosamples and Federated Cohort Data Access

Exposome research must establish relationships with other fields working in the areas of human health including biomedical and clinical communities who have established infrastructure resources at local, national, and international levels: for example, the International HundredK+ Cohorts Consortium (IHCC).^118^ This includes access to BioBanks and federated cohort data, including for LMICs. Infrastructures can support large-scale exposomics and omics approaches, data storage, and data analysis, and these are critical for the development of exposome research. In addition, the community must adopt and build on standards and frameworks defined by the Global Alliance for Genomics and Health (GA4GH)^119^ for the ethical and FAIR use of human genomic data.

### Moving toward Personalized Medicine, Population Health Risk Assessment, and One Health

Better integration of molecular methods in exposome science has potential impacts on both precision medicine and environmental health. The field must engage with key stakeholders in both to fully realize the benefits of exposome science. To achieve this, clinical studies that include exposomics information, in addition to other molecular data, should be encouraged, which could include specific funding agencies (e.g., National Institute of Environmental Health Sciences, Horizon Europe). Consortia including clinicians, exposome scientists, and biologists can achieve this. Such studies will support the transfer of personalized approaches into the clinics, taking into consideration environmental and occupational exposures.

## Summary

Mechanistic molecular techniques have real potential in defining the causative effects of the exposome on health. While the adoption of multiomics is beginning to become the norm across exposome cohorts, subsequent hypothesis-driven laboratory testing with support of relevant *in silico* approaches is not only necessary but should be prioritized to fully leverage the potential insights of exposure effects on phenotypes from the molecular scale.
